# Evaluating methods for risk prediction of Covid-19 mortality in nursing home residents before and after vaccine availability: a retrospective cohort study

**DOI:** 10.1186/s12874-024-02189-3

**Published:** 2024-03-27

**Authors:** Komal Aryal, Fabrice I. Mowbray, Anna Miroshnychenko, Ryan P. Strum, Darly Dash, Michael P. Hillmer, Kamil Malikov, Andrew P. Costa, Aaron Jones

**Affiliations:** 1 Department of Health Research Methods, Evidence, and Impact, McMaster University, 1280 Main St W, Hamilton, ON L8S 4L8 Canada; 2 ICES, Hamilton, ON Canada; 3 College of Nursing, Michigan State University, East Lansing, MI USA; 4 Institute of Health Policy, Management and Evaluation, University of Toronto, Toronto, Canada; 5 Capacity Planning and Analytics, Ontario Ministry of Health, Toronto, Canada

**Keywords:** Long-term care, Nursing home, Older adults, COVID-19, Prediction modeling, Cohort study, Machine learning, Electronic Health Record

## Abstract

**Background:**

SARS-CoV-2 vaccines are effective in reducing hospitalization, COVID-19 symptoms, and COVID-19 mortality for nursing home (NH) residents. We sought to compare the accuracy of various machine learning models, examine changes to model performance, and identify resident characteristics that have the strongest associations with 30-day COVID-19 mortality, before and after vaccine availability.

**Methods:**

We conducted a population-based retrospective cohort study analyzing data from all NH facilities across Ontario, Canada. We included all residents diagnosed with SARS-CoV-2 and living in NHs between March 2020 and July 2021. We employed five machine learning algorithms to predict COVID-19 mortality, including logistic regression, LASSO regression, classification and regression trees (CART), random forests, and gradient boosted trees. The discriminative performance of the models was evaluated using the area under the receiver operating characteristic curve (AUC) for each model using 10-fold cross-validation. Model calibration was determined through evaluation of calibration slopes. Variable importance was calculated by repeatedly and randomly permutating the values of each predictor in the dataset and re-evaluating the model’s performance.

**Results:**

A total of 14,977 NH residents and 20 resident characteristics were included in the model. The cross-validated AUCs were similar across algorithms and ranged from 0.64 to 0.67. Gradient boosted trees and logistic regression had an AUC of 0.67 pre- and post-vaccine availability. CART had the lowest discrimination ability with an AUC of 0.64 pre-vaccine availability, and 0.65 post-vaccine availability. The most influential resident characteristics, irrespective of vaccine availability, included advanced age (≥ 75 years), health instability, functional and cognitive status, sex (male), and polypharmacy.

**Conclusions:**

The predictive accuracy and discrimination exhibited by all five examined machine learning algorithms were similar. Both logistic regression and gradient boosted trees exhibit comparable performance and display slight superiority over other machine learning algorithms. We observed consistent model performance both before and after vaccine availability. The influence of resident characteristics on COVID-19 mortality remained consistent across time periods, suggesting that changes to pre-vaccination screening practices for high-risk individuals are effective in the post-vaccination era.

**Supplementary Information:**

The online version contains supplementary material available at 10.1186/s12874-024-02189-3.

## Introduction

The COVID-19 pandemic led to an exponential surge in the number of deaths within nursing homes (NH) [1–4]. In 2020, 63% of all deaths in the NH were attributed to COVID-19 among NH resident deaths in Canada [5]. NH residents have been disproportionately affected by COVID-19 illness due to their complex health and physical care needs, coupled with increasing fraility [6]. SARS-CoV-2 vaccines have been effective in reducing hospitalization, COVID-19 symptoms, and mortality for NH residents, and NH residents were prioritized during vaccine rollout [7–11]. Nearly 80% of NH residents had received one dose of a SARS-CoV-2 vaccine in Canada by the end of January 2021 [12]. Even after vaccination however, residents with high-risk profiles can still experience poor outcomes from COVID-19, including death [7]. Numerous resident characteristics (e.g., age, gender, cognitive status, and physical functioning) have been examined as prognostic factors for COVID-19 mortality [13, 14]. However, little is known about how the predictability of COVID-19 mortality changed due to the vaccine rollout.

Regression-based and tree-based machine learning models have been widely used in health and health services research. Advanced machine learning algorithms demonstrate remarkable capability in identifying high-risk subpopulations, particularly when predictors exhibit intricate interaction effects [15]. As a result, these methods have gained considerable popularity in research involving complex and vulnerable populations, such as NH residents. However, a unanimous consensus on the most suitable method to discriminate outcomes remains elusive, primarily due to the susceptibility of tree-based models to overfitting, which compromises the model’s generalizability [16–19]. Accurate mortality prediction at the individual NH resident level could greatly benefit healthcare professionals in prioritizing medical care and enabling efficient resource planning.

Our study aimed to utilize different machine learning methods to compare COVID-19 mortality prognostication in NH residents before and after vaccine availability. Our objectives were to establish the accuracy of various machine learning models, examine changes to model performance, and identify resident characteristics that have the strongest associations with 30-day COVID-19 mortality, before and after availability.

## Methods

### Study design

We conducted a population-based retrospective cohort study analyzing data from all NH facilities across Ontario, Canada. This study was reviewed and approved by the Hamilton Integrated Research Ethics Board (HiREB # 10,959-C). To ensure accurate reporting, we followed the Strengthening the Reporting of Observational Studies in Epidemiology (STROBE) statement guidelines for this cohort study.

### Data sources

Four population-level health administrative databases were examined. The Continuing Care Reporting System (CCRS) is a data repository of clinical assessments that are completed for each NH resident using the Resident Assessment Instrument - Minimum Data Set (RAI-MDS) 2.0 [20]. Residents receive a RAI-MDS assessment upon admission into the facility and then every three months thereafter. The CCRS also records changes to resident status such as discharges from NH, death died in the facility, or during a hold-bed period when a resident is temporarily elsewhere such as the hospital. The Ontario Laboratories Information System (OLIS) records lab test orders and results from hospitals, community labs and public health labs, including those for COVID-19, for which we used the specimen collection date. The Ontario Integrated Public Health Information System (IPHIS) documents public health cases, including COVID-19, for which we used the episode start date, and records dates of death if the resident died during the COVID-19 episode. We used the Discharge Abstract Database (DAD) to identify all those who were admitted to the hospital and recorded as deceased during their stay. Datasets used were linked and deidentified by the Ontario Ministry of Health (MOH).

### Study participants

We included all residents in our cohort who were admitted into the NH and stayed at an NH for one or more days between March 7, 2020, and July 31, 2021. We excluded residents if they had no complete RAI-MDS 2.0 assessment or resided outside of Ontario. For residents who were admitted to an NH more than once, we utilized the first assessment per time period to avoid correlated data among residents. Our study only utilized assessments completed on or before the positive SARS-CoV-2 infection date [21]. 

### Outcome measure & exposure periods

Our primary outcome was 30-day mortality following a laboratory-verified positive SARS-CoV-2 test. We examined positive cases during two time periods. The first time period lasted from the start of the pandemic to the beginning of vaccine availability (March 7, 2020, to December 31, 2020) which was selected based on the closest date to the first positive SARS-CoV-2 infection in NH and the first reported outbreak [22]. The second time period lasted from vaccine availability until the end of the study period (January 1, 2021, to July 31, 2021). We selected January 1, 2021 as our second time period because it approximates the start of vaccine rollout in the NH [23]. 

Residents in both time periods were followed for 30 days to measure the primary outcome. If residents tested positive for SARS-CoV-2 more than once in each study period, we included only the first instance to avoid correlated data. Patients who tested positive during the first 30 days of the second time period were only included if there was no documentation of a positive COVID-19 test in the previous 30 days.

### Resident characteristics

Resident characteristics for inclusion in the machine learning models were selected based on their availability in our data sources, clinical expertise and prior literature [3, 24–28]. Resident characteristics came from the completed MDS 2.0 assessments. These characteristics include demographic, clinical, and social characteristics reported at the NH facility that are expected to be associated with COVID-19 mortality. The methods employed for resident character selection in the study were not statistically driven and are described in detail in our previous work [29]. 

### Statistical analysis

Descriptive statistics were reported using measures of frequency and central tendency to compare residents who died due to COVID-19 both before and after vaccine availability. Variable selection was performed a priori using both theoretical and clinical methods based on existing literature [29]. We used five machine learning algorithms to predict COVID-19 death. These included logistic regression, LASSO logistic regression, classification and regression tree (CART), random forests, and gradient boosted trees (GBT). Data were screened for the presence and pattern of missingness. Only five (0.03%) cases of missing data were present for one predictor variable and these cases were deleted within each analysis.

We performed hyperparameter tuning for each non-logistic regression model to determine the optimal set of parameters for each machine learning model. Tuning was performed using 10-fold cross-validation with a 1000-iteration random grid search over the parameter space, selecting the parameters that maximized the area under the receiver operating characteristic curve (AUC). All tuning and testing were performed independently for the two vaccination periods. Final performance was determined by calculating the AUC for each model using an independent and identical 10-fold cross-validation and by computing F1 scores and balanced accuracy. Permutation methods were used to calculate variable importance by repeating (*n* = 50) randomly permutated values of each predictor in the dataset and re-evaluating the model’s performance, which was not nested inside the cross-validation [30]. The average difference in the AUC was used to measure variable importance, with negative values of greater magnitude indicating more importance. Model calibration was assessed visually using calibration plots. Data were managed and analyzed using R-Studio 4.2.2.

### Sensitivity analysis

We conducted a sensitivity analysis by excluding January 2021, during which time the first dose was being rolled-out to NH residents. We recalculated performance statistics for logistic regression and LASSO regression. Since fewer infections occurred between February 2021 to July 2021, complex machine learning models including random forests, CART, and GBT were not conducted in the sensitivity analysis.

## Results

There were 14,977 NH residents who tested positive for a COVID-19 infection during the study period. A total of 11,291 NH residents tested positive for COVID-19 before vaccine availability and 3686 NH residents tested positive after vaccine availability. The median time (25th -75th percentiles) from the MDS 2.0 assessment date to the COVID-19 date was 44 days (22–67).

Table 1 displays a comprehensive list of resident characteristics for NH residents who were diagnosed with COVID-19 before and after vaccine availability. Most residents were female (65.8%; 67.6%), 65 + years (92.6%; 93.6%), and were diagnosed with dementia (52.8%; 52.7%) in the before and after vaccine availability groups, respectively. There were 2937 (26.0%) NH residents who died before vaccine availability and 727 (19.7%) NH residents who died after vaccine availability.

**Table 1:** Descriptive Characteristics of Nursing Home Residents who were Diagnosed with COVID-19 Infection

Resident Characteristic	Before Vaccine Availability Mar 7, 2020 – Dec 31, 2020 (*n* = 11,291) n(%)	After Vaccine Availability Jan 1, 2021– July 31, 2021 (*n* = 3686) n(%)
**Age Group**, years		
<64	832 (7.4)	235 (6.4)
65–74	1466 (13.0)	481 (13.0)
75–84	3134 (27.8)	994 (27.0)
85+	5859 (51.9)	1976 (53.6)
**Sex**		
Male	3857 (34.2)	1195 (32.4)
Female	7434 (65.8)	2491 (67.6)
**Comorbidities**		
Cardiovascular Diseases	2771 (24.5)	898 (24.4)
Congestive Heart Failure	429 (3.8)	183 (5.0)
Diabetes	3431 (30.4)	1128 (30.6)
Respiratory Disease^a^	935 (8.3)	418 (11.3)
Renal Failure	399 (3.5)	140 (3.8)
Dementia	5958 (52.8)	1942 (52.7)
Depression	3576 (31.7)	1229 (33.3)
Anxiety	462 (4.1)	177 (4.8)
Cancer	320 (2.8)	120 (3.2)
Fever and Headache	353 (3.1)	120 (3.2)
**Polypharmacy**		
Taking 5 or more medications/daily	10,417 (92.3)	3444 (93.4)
Taking less than 5 medications/daily	874 (7.7)	242 (6.6)
**Nutrition Risk** ^**b**^	3955 (35.0)	1504 (40.8)
**Daily Decision Making**		
Independent	914 (8.1)	436 (11.8)
Modified Independence	2283 (20.2)	780 (21.2)
Moderately Impaired	5679 (50.3)	1775 (48.2)
Severely Impaired	2410 (21.3)	695 (18.9)
**Activities of Daily Living**		
Independent or Supervision Required	501 (4.4)	170 (4.6)
Limited	734 (6.5)	226 (6.1)
Extensive	3046 (27.0)	993 (27.0)
Maximal	3014 (27.7)	921 (25.0)
Dependence	2872 (25.4)	990 (26.8)
Total Dependence	1123 (9.9)	386 (10.5)
**Cognitive Performance Scale (CPS)**		
Intact	1651 (14.6)	658 (17.8)
Moderate	7224 (64.0)	2333 (63.3)
Severe	2415 (21.4)	695 (18.9)
**Pain**		
Less than Daily	1742 (15.4)	671 (18.2)
Daily but Not Severe	279 (2.5)	134 (3.6)
Daily and Severe	56 (0.5)	22 (0.6)

^a^COPD, Emphysema, Asthma, & Dyspnea. ^b^Decreased appetite, weight loss, & dehydration

### Model performance

Table 2 displays the final model performance from the five machine learning models. The cross-validated AUC performance ranged from 0.64 to 0.67, irrespective of vaccination period. Across all models, GBT had the highest discrimination ability with an AUC of 0.67 for both the before and after vaccination periods, although the discriminative accuracy was not significantly different from the other machine learning models, excluding CART (*p* < .05). Our CART model had the lowest discrimination ability both before and after the vaccination periods compared to logistic regression, random forests, and gradient-boosted trees. F1 scores and balanced accuracy are reported in Appendix A and model calibration are presented in Appendix B. The range of model parameters are reported in Appendix C.

**Table 2:** Final Model Performance of Logistic and Machine Learning Models Before and After Vaccine Availability

Model Type	Before Vaccine Availability Mar 7, 2020 – Dec 31, 2020 (*n = 11,291*)	After Vaccine Availability Jan 1, 2021– July 31, 2021 (*n = 3686*)
Logistic Regression	0.670	0.670
Lasso Regression	0.664	0.664
Random Forest	0.662	0.663
Classification and Regression Tree (CART)	0.641	0.646
Gradient Boosted Trees	0.672	0.670

### Associations with COVID-19 mortality

**Fig. 1:**
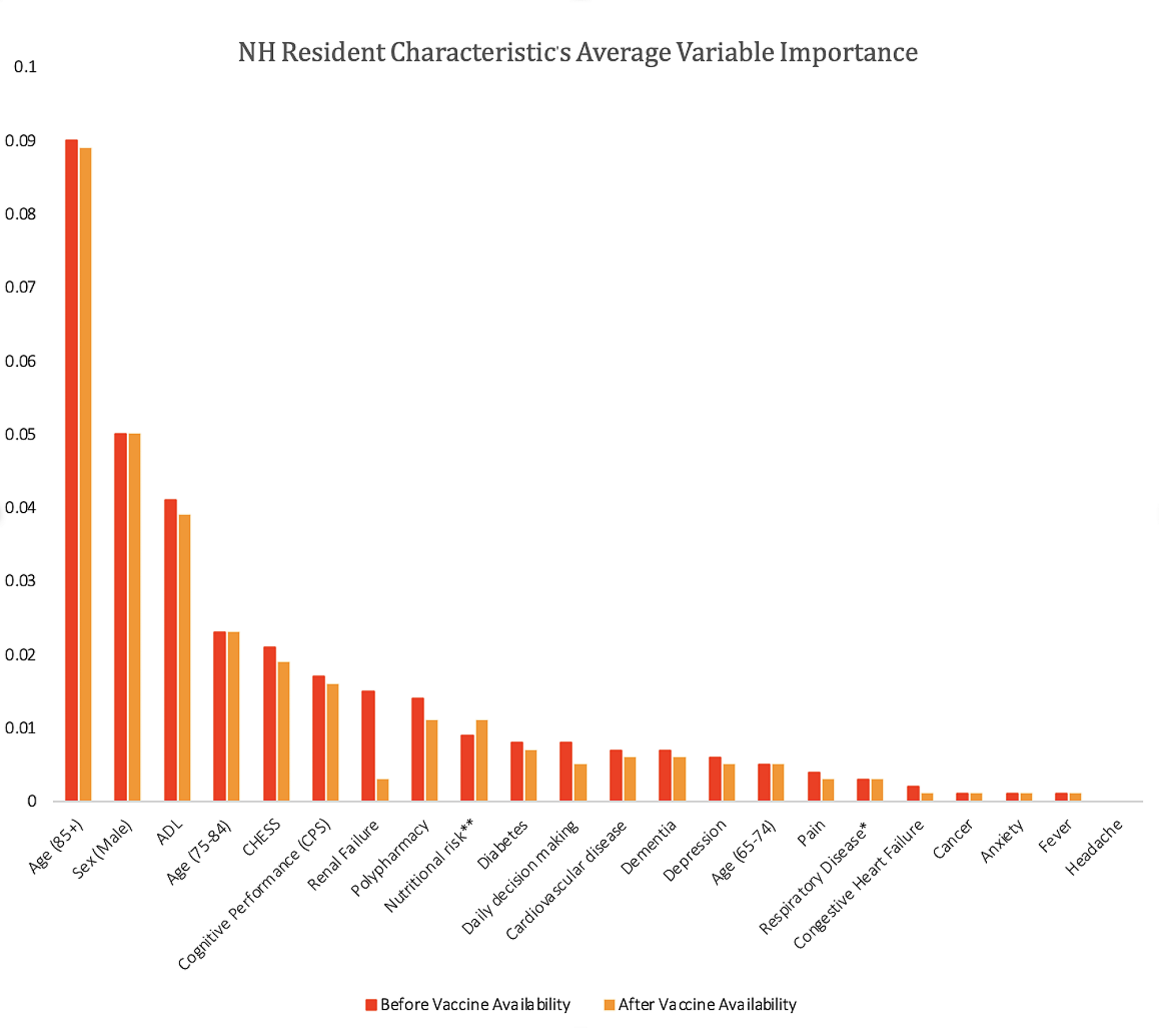
The Average Inverse Variable Importance for all Resident Characteristics Before and After Vaccine Availability. ^*^Respiratory Disease: Chronic Obstructive Pulmonary Disease, Emphysema, Asthma, & Dyspnea ^**^Nutrition Risk: Decreased appetite, weight loss, & dehydration

Approximately half (11/20) of the resident characteristics contributed to the performance of all five models (Appendix D). On average, the five most influential resident characteristics both before and after vaccine availability were being 85 years older, being aged 75–84, being male, deteriorating ADLs, and having a high score on the CHESS scale (i.e., health instability) (Fig. 1). The least influential resident characteristics across all five models both before and after vaccine availability, having a headache, having a fever, experiencing anxiety, having cancer, congestive heart failure, and having respiratory disease. Overall, there was little difference in the variable importance before and after vaccine availability and between the five models.

### Sensitivity analysis

There were significantly fewer cases in the February 1, 2021, to July 31, 2021 (*n* = 828) than January 1, 2021 to July 31, 2021 (*n* = 3686). Analysis showed similar but slightly higher AUC values for logistic regression before (0.68) and after (0.69) vaccine availability. Similarly, the AUC values for LASS0 regression before (0.66) and after (0.68) were slightly higher after vaccine availability.

## Discussion

We used one statistical model and four machine learning models to examine 30-day COVID-19 mortality among NH residents before and after vaccine availability in Ontario, Canada. All models exhibited similar predictive accuracy both between models and across the COVID-19 vaccine availability time periods. Approximately half of resident characteristics were informative in identifying residents at high-risk of COVID-19 mortality. These factors remained consistent regardless of vaccine availability in all models.

Our study highlights a ceiling effect on the discriminative ability of machine learning algorithms when using routinely collected administrative data compared to the statistical logistic regression model. While GBT models can accommodate complex patterns within the data, they are computationally complex, and their “black box” nature makes them less appealing to clinical audiences. Prior works have demonstrated similar discriminative accuracies between GBT and logistic regression in complex older adults [30–32]. Further, our study contributes evidence to prior work demonstrating that the use of complex supervised machine learning algorithms is unlikely to out-perform standard regression models using highly structured data [33]. We conducted a comparative analysis of the discriminability of machine learning methods before and after COVID-19 vaccine availability. We found no discernible difference in the performance of these models based on vaccine availability.

Previous studies have reported higher AUC values when predicting 30-day COVID-19 mortality [34]. However, many of these studies focused on 30-day COVID-19 mortality within broad populations, in which age is a highly discriminative predictor of mortality, a consistent result of our study. For example, a study by Hippisley-Cox et al., [26] focused on a broad population of all adults in England and did not assess the risk of COVID-19 mortality for the NH population. In contrast, our study specifically aims to predict COVID-19 mortality among older, frail nursing home residents. The limited heterogeneity in our samples makes it more challenging to discern individuals who are at a higher risk of death.

We sought to evaluate whether the significance and magnitude of the resident characteristics in these models differed between vaccine availability periods. The important resident characteristics in our model were older age, male sex, and deteriorating ADL status with age being the most influential. However, our results indicate that there is little difference in resident characteristics influencing COVID-19 mortality based on vaccine availability. NH resident characteristics alone were not sufficiently able to determine which residents were at greatest risk of COVID-19 mortality, as evidenced by their relatively weak AUC values, irrespective of time period.

From our research, it is evident that pre-vaccination prognostic scores and models are still informative of post-vaccination scores and models could be effective when employed post vaccination rollout. Existing practices to identify residents at high-risk of COVID-19 death likely do not need to be adjusted. This finding can help determine future care plans for both vaccinated and unvaccinated NH populations. Future studies should determine if COVID-19 risk factors remain stable for older individuals living in congregate care settings such as retirement homes.

### Limitations

We leveraged a population-level database of all NH residents across Ontario and reported on a wide array of resident factors and geriatric syndromes known to be prognostic of mortality post-SARS-CoV-2 infection. However, we were limited to secondary data collected across databases. Undocumented resident characteristics, such as ethnicity or race, may influence mortality but are not recorded in the RAI-MDS 2.0. Our databases did not capture the accurate date or type of vaccine received by NH residents and thus were unable to stratify based on actual vaccination type. These predictors may have been informative in predicting COVID-19 mortality, but we were unable to include them in our model. Our analysis began during the early stages of COVID-19 and some residents may not have had a COVID-19 test before dying results in some COVID-19 deaths may not being captured. The discriminative accuracy of statistical models was *fair* despite having a panel of prognostic factors known to influence 30-day COVID-19 mortality. However, the use of prognostic models with this level of discriminative ability in population-level research is common, considering the difficulties of predicting within a complex-adaptive system [35, 36]. 

### Conclusions and implications

Our study determined that all statistical and machine learning algorithms examined displayed similar predictive accuracy. This suggests that there would be no benefit in choosing a more complex tree-based model over standard regression for these data sources. Overall, the performance of the models did not differ before and after vaccine availability, indicating that vaccine uptake did not change COVID-19 mortality prognostication. Resident characteristics influencing 30-day COVID-19 mortality are similar both before and after vaccine availability. The stability of risk factors and performance between vaccination periods suggests that models generated to predict COVID-19 mortality pre-vaccination are valid for use in the post-vaccination era.

### Electronic supplementary material

Below is the link to the electronic supplementary material.

Supplementary Material 1

## Data Availability

Data access is governed separately by the Ontario Personal Health Information Protection Act and held securely at McMaster University. Analytic coding is available upon request from the authors with appropriate approvals to protect the security of source the data architecture.
