# Supplementary material for: Evaluating methods for risk prediction of Covid-19 mortality in nursing home residents before and after vaccine availability: a retrospective cohort study
Source: BMC Med Res Methodol. 2024 Mar 27;24:77. doi: 10.1186/s12874-024-02189-3 (PMC10976701; doi:10.1186/s12874-024-02189-3)
Supplement: Supplementary file 1 — Supplementary Material 1 [file 12874_2024_2189_MOESM1_ESM.docx]

***Appendix***

***Appendix A:*** *Final Model Performance Using F1 Score and Balanced Accuracy of Logistic and Machine Learning Models Before and After Vaccine Availability*

| **Model Type** | **Before Vaccine Availability**  Mar 7, 2020 – Dec 31, 2020  (*n = 11,291*) | | **After Vaccine Availability**  Jan 1, 2021– July 31, 2021  (*n = 3,686*) | |
| --- | --- | --- | --- | --- |
|  | **F1 Score** | **Balanced Accuracy** | **F1 Score** | **Balanced Accuracy** |
| **Logistic Regression** | 0.159 | 0.534 | 0.0269 | 0.506 |
| **Lasso Regression** | 0.471 | 0.632 | 0.390 | 0.619 |
| **Random Forest** | 0.124 | 0.521 | 0.122 | 0.501 |
| **Classification and Regression Tree (CART)** | 0.117 | 0.523 | 0.105 | 0.519 |
| **Gradient Boosted Trees** | 0.103 | 0.517 | 0.109 | 0.518 |

***Appendix B:*** Calibration Plots for All Model Before and After Vaccination

***Appendix BA:*** Calibration Plot for Logistic Regression Before Vaccination


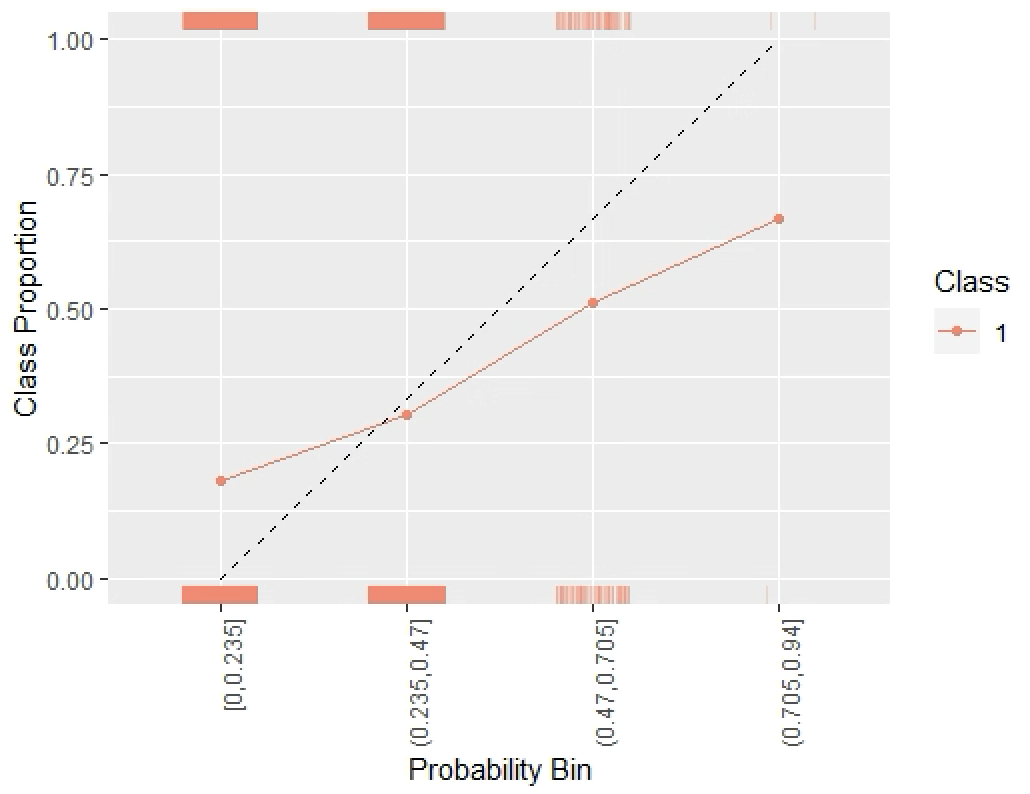


***Appendix BB:*** Calibration Plot for Logistic Regression After Vaccination


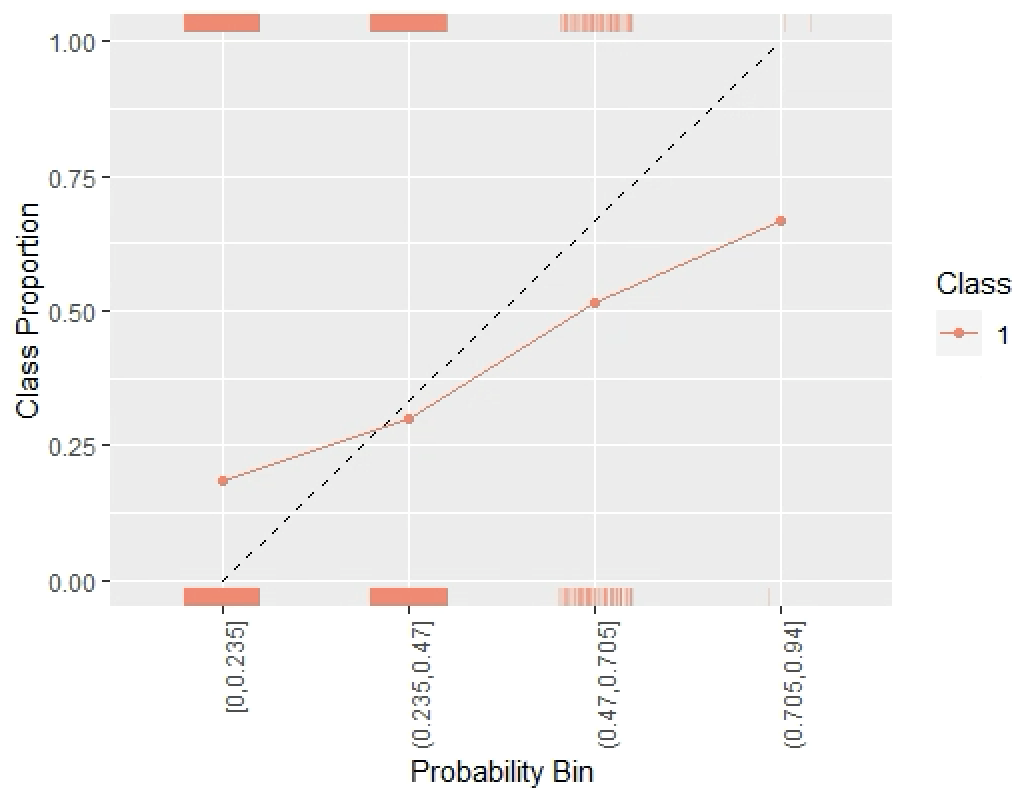


***Appendix BC:*** Calibration Plot for LASSO regression Before Vaccination


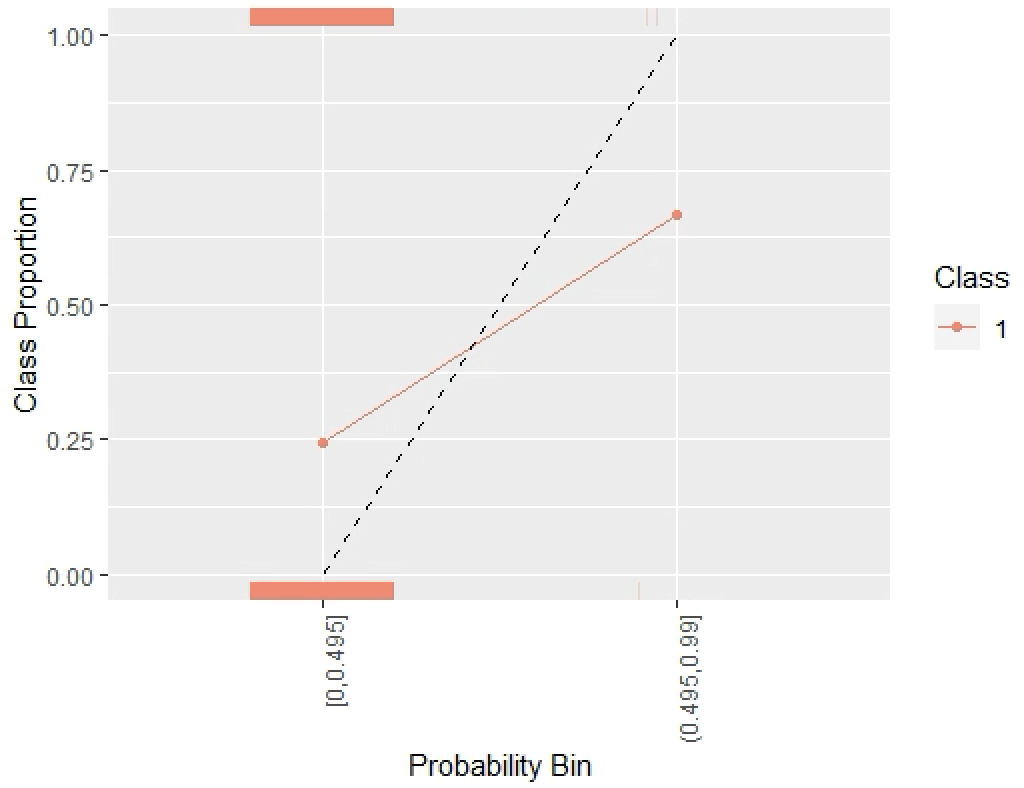


***Appendix BD:*** Calibration Plot for LASSO regression After Vaccination


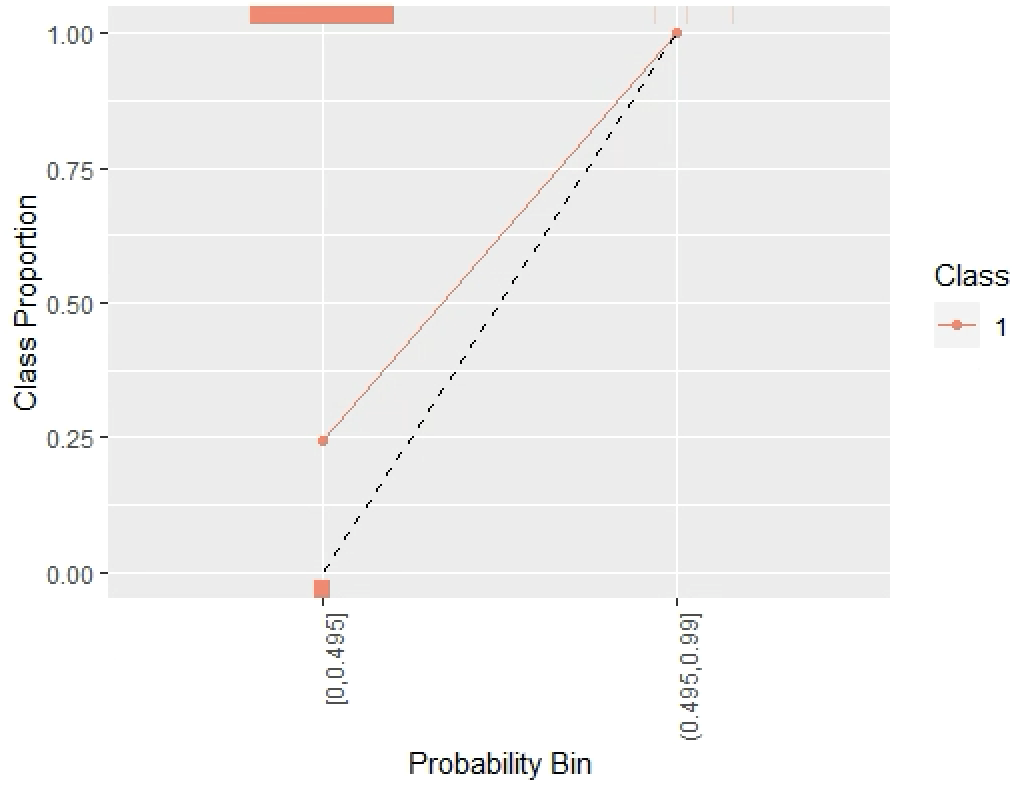


***Appendix BE:*** Calibration Plot for Random Forest Before Vaccination


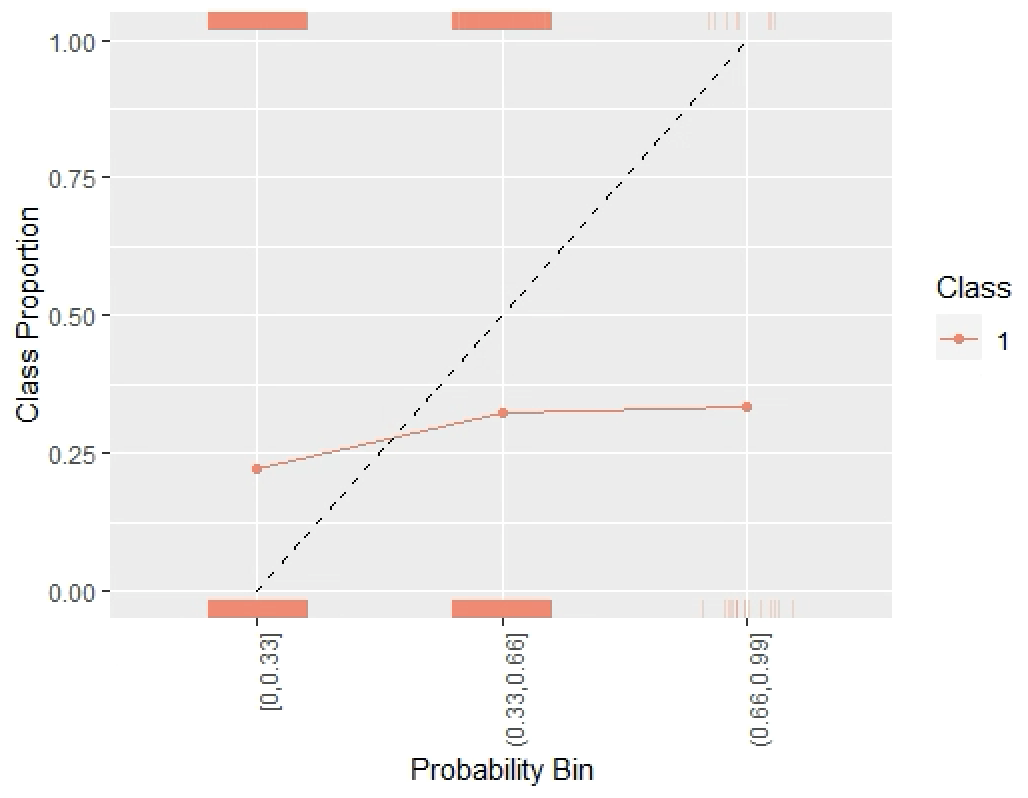


***Appendix BF:*** Calibration Plot for Random Forest After Vaccination


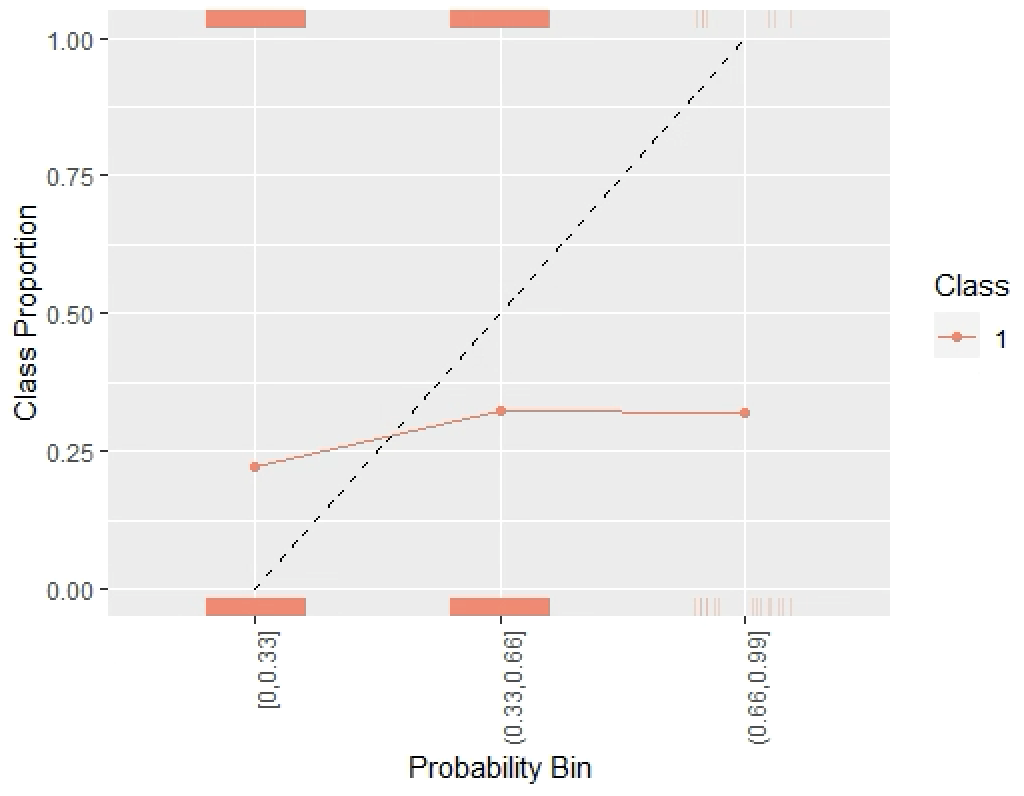


***Appendix BG:*** Calibration Plot for Classification and Regression Trees Before Vaccination


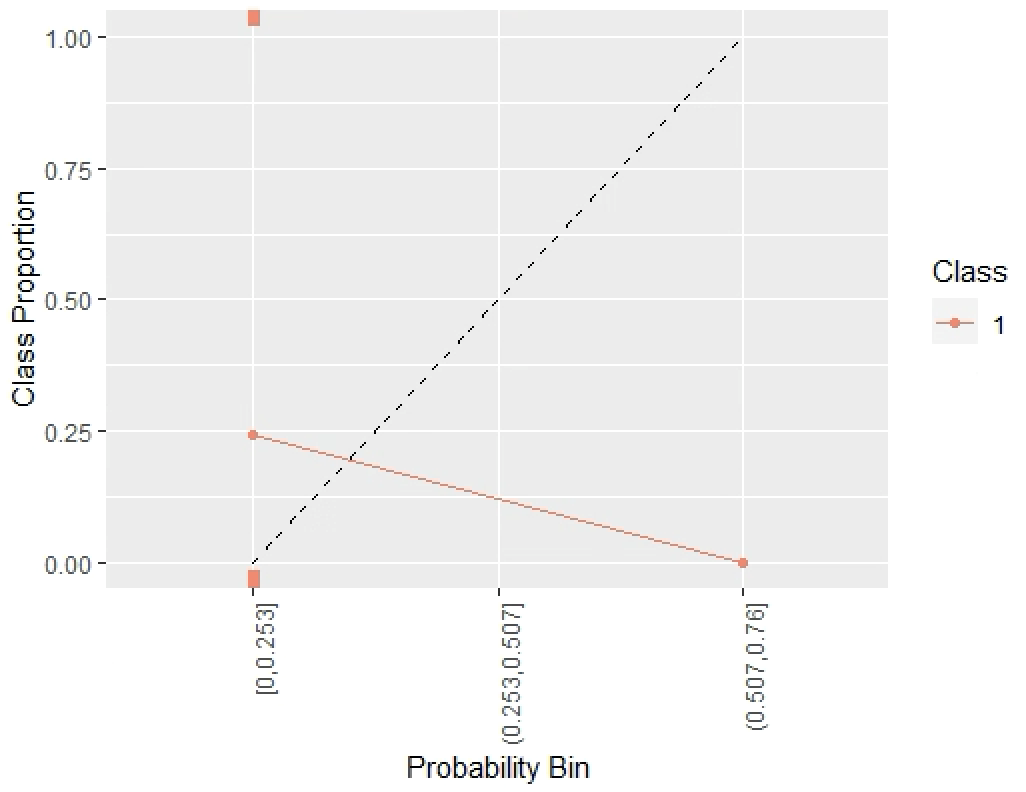


***Appendix BH:*** Calibration Plot for Classification and Regression Trees After Vaccination


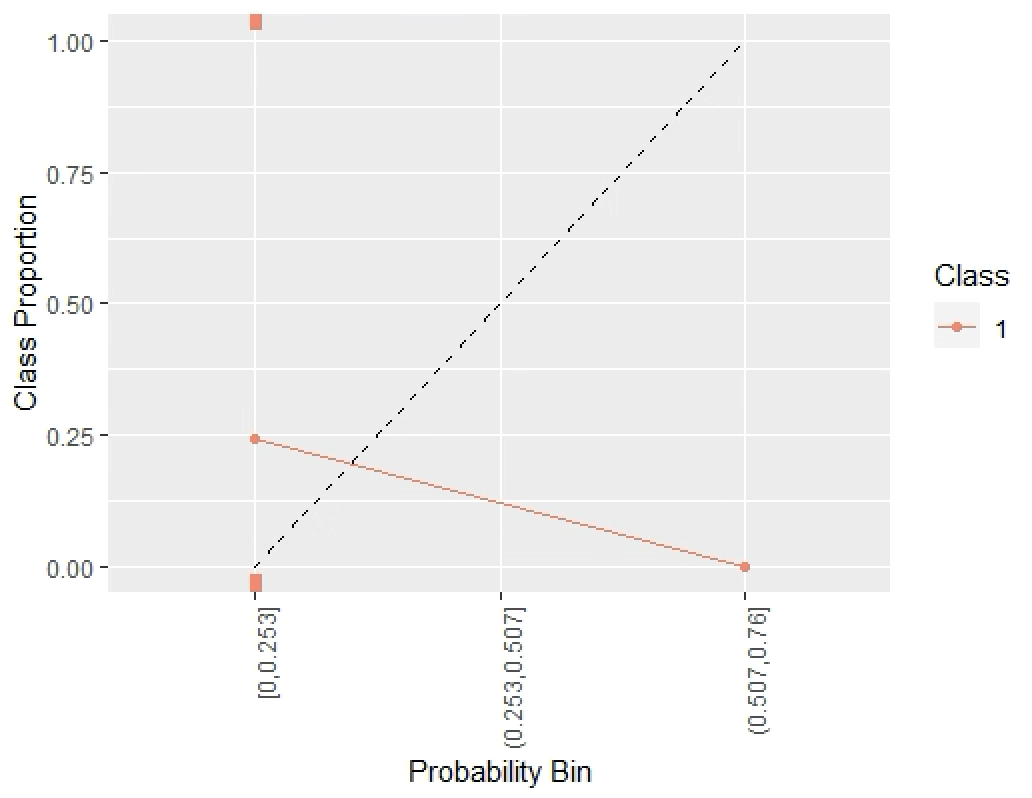


***Appendix BI:*** Calibration Plot for Gradient Boosted Trees Before Vaccination


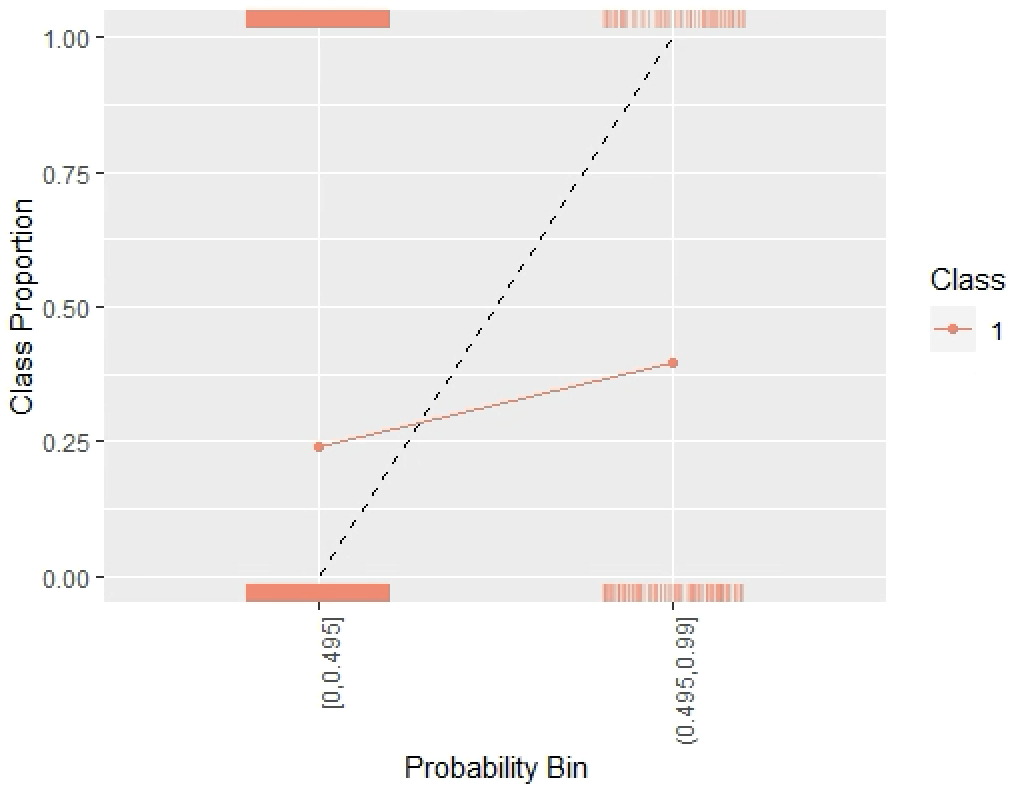


***Appendix BJ:*** Calibration Plot for Gradient Boosted Trees After Vaccination


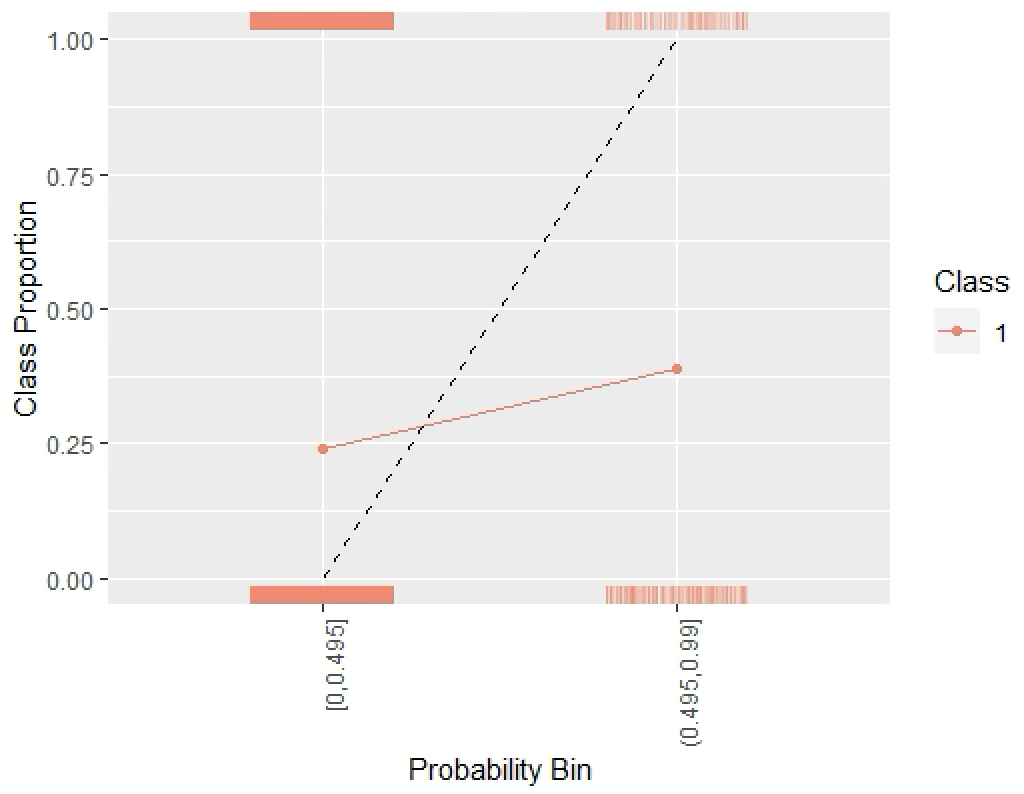


***Appendix C: Details of Optimal Parameters for all the Machine Learning Models***

| **Machine Learning Method** | | **Details of hyperparameter tuning** |
| --- | --- | --- |
|  | ***Parameter Name*** | ***Range*** |
| **Gradient Boosted Trees** | **Learning Rate (eta)** | 10^-3.3 to 10^-1 |
|  | **Nrounds** | 300 to 1000 |
|  | **Max Depth** | 3 to 15 |
| **CART** | **Complexity parameter** | 10^-6 to 10^-1 |
|  | **Min Bucket** | 5, 10, 15, and 25 |
| **Random Forest** | **Number of Trees** | 500 |
| **LASSO** | Not Applicable | |
| **Logistic** | Not Applicable | |

***Appendix D:*** The Variable Importance for all Models Pre-and Post-vaccination

| **Variables** | **Logistic** | | **Lasso** | | **Random Forest** | | **Classification Trees** | | **Gradient Boosted Trees** | |
| --- | --- | --- | --- | --- | --- | --- | --- | --- | --- | --- |
|  | **Before** | **After** | **Before** | **After** | **Before** | **After** | **Before** | **After** | **Before** | **After** |
| **Age (65-74)** | -0.007 | -0.007 | 0 | 0 | -0.0137 | -0.011 | -0.005 | -0.005 | -0.003 | -0.001 |
| **Age (75-84)** | -0.040 | -0.040 | -0.009 | -0.011 | -0.0222 | -0.021 | -0.029 | -0.027 | -0.021 | -0.014 |
| **Age (85+)** | -0.120 | -0.120 | -0.073 | -0.075 | -0.0727 | -0.070 | -0.109 | -0.102 | -0.093 | -0.076 |
| **Sex (Male)** | -0.042 | -0.042 | -0.041 | -0.042 | -0.0661 | -0.064 | -0.057 | -0.055 | -0.055 | -0.046 |
| **Polypharmacy** | -0.002 | -0.002 | -0.001 | -0.001 | -0.0386 | -0.036 | -0.017 | -0.009 | -0.014 | -0.010 |
| **Cardiovascular disease** | -0.005 | -0.005 | -0.002 | -0.001 | -0.0202 | -0.018 | -0.003 | -0.001 | -0.003 | -0.004 |
| **Congestive Heart Failure** | -0.000* | -0.000* | 0 | 0 | -0.0058 | -0.005 | -0.001 | 0 | -0.001 | -0.001 |
| **Diabetes** | -0.003 | -0.003 | -0.003 | -0.003 | -0.0257 | -0.023 | -0.004 | -0.003 | -0.005 | -0.005 |
| **Renal Failure** | -0.003 | -0.003 | -0.001 | -0.001 | -0.0066 | -0.006 | -0.002 | -0.002 | -0.003 | -0.002 |
| **Respiratory Disease^a^** | -0.001 | -0.001 | -0.000* | -0.000* | -0.01097 | -0.009 | -0.002 | -0.002 | -0.003 | -0.002 |
| **Dementia** | -0.000* | -0.000* | -0.000* | -0.000* | -0.0271 | -0.025 | -0.006 | -0.005 | -0.002 | -0.001 |
| **Cancer** | -0.000* | -0.000* | 0 | 0 | -0.00413 | -0.004 | 0 | 0 | -0.001 | -0.000* |
| **Anxiety** | -0.000* | -0.000* | 0 | 0 | -0.00511 | -0.005 | 0 | 0 | -0.001 | -0.000* |
| **Depression** | -0.001 | -0.001 | 0 | 0 | -0.0231 | -0.021 | -0.004 | -0.003 | -0.002 | -0.002 |
| **Daily decision making** | -0.000* | -0.000* | 0 | 0 | -0.0237 | -0.022 | -0.016 | -0.001 | -0.003 | -0.002 |
| **Fever** | -0.000* | -0.000* | 0 | 0 | -0.00383 | -0.003 | 0 | 0 | -0.001 | -0.000* |
| **Headache** | -0.000* | - 0.000* | 0 | 0 | -0.00134 | -0.001 | 0 | 0 | -0.000* | -0.000* |
| **ADL** | -0.020 | -0.0198 | -0.023 | -0.023 | -0.0645 | -0.060 | -0.061 | -0.056 | -0.047 | -0.035 |
| **Pain** | -0.000* | -0.0001 | 0 | 0 | -0.01623 | -0.014 | -0.002 | -0.001 | -0.002 | -0.001 |
| **Cognitive Performance (CPS)** | -0.013 | -0.013 | -0.010 | -0.010 | -0.035597 | -0.033 | -0.015 | -0.013 | -0.013 | -0.012 |
| **CHESS** | -0.013 | -0.013 | -0.022 | -0.021 | -0.03 | -0.029 | -0.023 | -0.020 | -0.017 | -0.013 |
| **Nutritional risk^b^** | -0.001 | -0.001 | 0 | 0 | -0.02375 | -0.035 | -0.015 | -0.015 | -0.004 | -0.003 |

^a^COPD, Emphysema, Asthma, & Dyspnea

^b^Decreased appetite, weight loss, & dehydration

*Reported value is between 0 and -0.0005

***Appendix E:*** The Mean and Standard Deviation for all Continuous Variables in the Model Before and After Vaccination

| Variable Name | | Before Vaccination | After Vaccination |
| --- | --- | --- | --- |
| Polypharmacy | Mean | 10.3 | 10.8 |
|  | Standard Deviation | 4.5 | 4.6 |
